# Effects of Psychedelics in Older Adults: A Prospective Cohort Study

**DOI:** 10.1016/j.jagp.2024.05.007

**Published:** 2024-05-19

**Authors:** Hannes Kettner, Leor Roseman, Adam Gazzaley, Robin L. Carhart-Harris, Lorenzo Pasquini

**Affiliations:** Department of Neurology, Neuroscape, University of California, San Francisco, CA; Centre for Psychedelic Research, Department of Brain Sciences, Faculty of Medicine, Imperial College, London, UK; Department of Psychology, University of Exeter, Exeter, UK; and the Department of Psychiatry, University of California-San Francisco, San Francisco, CA.

**Keywords:** Aging, psychedelics, mental health, well-being, cohort study, naturalistic research

## Abstract

**Objective:**

Affective symptoms such as anxiety, low mood, and loneliness are prevalent and highly debilitating symptoms among older adults (OA). Serotonergic psychedelics are currently investigated as novel interventions for affective disorders, yet little is known regarding their effects in OA. We investigated the mental health effects and psychological mechanisms of guided psychedelic group experiences in OA and a matched sample of younger adults (YA).

**Methods:**

Using a prospective observational cohort design, we identified 62 OA (age ≥60 years) and 62 matched YA who completed surveys two weeks before, a day, two weeks, four weeks, and six months after a psychedelic group session. Mixed linear regression analyses were used to investigate longitudinal well-being changes, as well as baseline, acute, and post-acute predictors of change.

**Results:**

OA showed post-psychedelic well-being improvements similar to matched YA. Among baseline predictors, presence of a lifetime psychiatric diagnosis was associated with greater well-being increases in OA (B = 6.72, p = .016 at the four-week key-endpoint). Compared to YA, acute subjective psychedelic effects were less intense in OA and did not significantly predict prospective well-being changes. However, relational experiences before and after psychedelic sessions emerged as predictors in OA (r(36) = .37,p = 0.025).

**Conclusions:**

Guided psychedelic group sessions enhance well-being in OA in line with prior naturalistic and controlled studies in YA. Interestingly, acute psychedelic effects in OA are attenuated and less predictive of well-being improvements, with relational experiences related to the group setting playing a more prominent role. Our present findings call for further research on the effects of psychedelics in OA.

## INTRODUCTION

Emotions are central to human functioning, guiding thought and action from the earliest to the latest days of life.^1^ Emotional experiences change over the adult life span, with older adults (OA) shifting their motivational goals towards optimizing emotional regulation and reporting positive emotions more often than their younger counterparts.^2^ However, affective symptoms, such as anxiety, mood instability, loneliness, and apathy, are common among OA and may herald incipient neuropsychiatric and neurological disorders, such as late-life depression and Alzheimer’s disease.^3-7^ In particular, loneliness, or the subjective feeling of being socially isolated, has been identified as a major modifiable risk factor for cognitive decline and worsening of mental well-being in OA.^5,6,8^ Loneliness has been shown to spread among social networks, to predict low life satisfaction, depressive symptoms, cognitive impairments, and Alzheimer’s disease dementia,^9^ highlighting the importance of nurturing healthy social connections in the elderly. Crucially, there is convergent evidence that conventional antidepressants—including selective serotonin reuptake inhibitors are less effective in treating affective symptoms in OA patient populations.^10,11^ On the contrary, their use has been associated with an increased incidence of adverse respiratory and gastrointestinal events, as well as emotional blunting when compared to placebo,^10-12^ highlighting the need for improved treatments for affective symptoms in OA.

Serotonergic psychedelics, such as psilocybin (contained in “magic mushrooms and truffles”), lysergic acid diethylamide (LSD), N, N-Dimethyltryptamine (DMT, the main ingredients of the Amazonian brew ayahuasca), and mescaline, have recently garnered increasing research interest, following several clinical trials suggesting the therapeutic potential for these substances in the treatment of affective symptoms across various neuropsychiatric disorders.^13^ Acute psychedelic effects are induced though the strong affinity of these substances for the serotonin 2A receptor;^14,15^ they include an altered state of consciousness characterized by intensified affect, vivified imagination and imagery, multisensory changes in perception, distorted sense of time, perceived spiritual and mystical experiences, and facilitated psychological insight. These latter effects, especially, have been indicated as mediators of therapeutic responses to psychedelics.^16-19^

Importantly, a recent review found that among 1,400 participants enrolled in 36 psychedelic trials since 1967, only 19 participants (1.4%) were 65 years or older.^20^ The safety and efficacy of psychedelic treatments in older populations thus remains largely unknown,^21^ although several authors have argued for the potential of psychedelics to loosen cognitive habits in old age generally^22^ as well as more formally as treatments for Alzheimer’s disease,^23-26^ including mild cognitive impairment^27^ and even healthy cognitive decline and age-related affective changes.^28,29^ Initial clinical trials are currently underway investigating the effects of psychedelics on affective symptoms in patients with Alzheimer’s disease (NCT04123314) and Parkinson’s disease (NCT04932434).

Outside of the highly controlled environment of clinical trials, psychedelic substances are commonly consumed recreationally, and increasingly also for self-medicative purposes.^30-32^ This includes guided individual and group settings, sometimes referred to as “ceremonies,” facilitated both underground and, in some countries, legally, e.g., at psychedelic retreats,^33^ where emphasis is placed on curating social contexts that foster interpersonal trust, openness, and expression of vulnerability.^34^ This is typically achieved through encouraging the structured sharing of personal and subjective experiences in group circles before and after psychedelic sessions and by exercising compassionate non-judgmental listening, reflecting some of the principles also employed in group psychotherapy.^35-37^ As such, previous work has shown that psychedelic group settings can enhance psychological well-being and social connectedness by generating a sense of togetherness and self-disclosure within and outside of the acute substance sessions.^34^

Prospective observational studies of group ceremonies and other naturalistic psychedelic use can therefore be used to monitor psychological outcomes among psychedelic users in a more ecologically valid fashion and in more diverse populations. For example, a recent study following this approach has shown improved well-being among young adult (YA) and adolescent psychedelic users, as well as age-dependent differences in salutogenic mechanisms^38^ and increased risk of adverse side effects.^39^ Yet, little is known regarding the potential of psychedelic experiences for improving mental health among OA. To investigate this question, we leveraged self-report data from an observational prospective cohort study of participants attending guided psychedelic group sessions (reported on also in^34^).

## MATERIALS AND METHODS

### Study Design and Participants

The present study employed a prospective cohort design utilizing an online convenience sample of individuals planning to attend an organized psychedelic retreat or group-based guided psychedelic ceremony session, on their own accord. Recruitment took place via two routes: firstly, through online advertisements on psychedelic-related social media channels (Facebook groups, Twitter), email newsletters, and online forums (e.g., Reddit), and secondly, through retreat facilitators who advertised the study to their prospective clients. Participants were able to review study information online, provided informed consent, and subsequently completed surveys through multiple e-mails sent before and after their planned experience: (1) Within two weeks prior to the session, assessing demographics and baseline scores of mental health related outcome variables; (2) 24 h after the session, assessing acute subjective effects; (3) one day after leaving the ceremony or retreat location, including variables related to the overall experience; and (4) two weeks, four weeks, and six months after the experience, measuring changes in the outcome variables. Eligibility criteria included being ≥18 years old, a good comprehension of the English language, and intentions to attend a psychedelic ceremony (i.e., involving use of psilocybin/magic mushrooms/truffles, LSD, ayahuasca, DMT/5-MeO-DMT, mescaline, or iboga/ibogaine). The study was approved by the Imperial College London’s Research Ethics Committee (ICREC) and the Joint Research Compliance Office (JRCO). For a full overview of the study design, see.^34^

Participants were included if they had completed at least the baseline and the 24h post-session survey. OA were identified based on a reported age of ≥60 and a set of matched YA was selected using nearest neighbor matching via the *MatchIt* package implemented in R (https://cran.r-project.org/web/packages/MatchIt/vignettes/MatchIt.html), matched for gender, education, psychiatric history, previous psychedelic use, baseline well-being, and drug dose. By projecting the above variables in a multidimensional Euclidean space, MatchIt uses propensity scores based on k-nearest distance between given data points to pair participants across the OA and YA samples. Through this procedure, MatchIt can improve parametric statistical models for estimating treatment effects in observational studies and reduce model dependence. As a non-parametric one-to-one matching algorithm, it implements the suggestions of Ho et al.,^40^ proposing matching as a non-parametric preprocessing procedure reducing the dependence of subsequent parametric models on specific distributional assumptions and improving the validity of causal inference. This procedure effectively allows researchers to use the same parametric analyses following matching as would be done without matching. Accordingly, parametric estimation-based regression models were run as normal, while both paired and independent-samples tests are presented for comparisons of means between the age groups, taking into consideration the recommendation by Austin^41^ of treating paired samples as dependent data.

### Measures

#### Baseline predictors.

At baseline, age, gender, education, extent of prior experience with psychedelic substances, expectations regarding potential beneficial effects of the experience (0–100 visual analogue scale), and self-reported history of psychiatric diagnoses were assessed, as well as basic information regarding the planned experience, such as substance type and location.

#### Outcome measures:

The Warwick-Edinburgh Mental Wellbeing Scale (WEMWBS)^42^ was assessed at baseline and at the three endpoints, namely two weeks, four weeks, and six months following the session to measure changes in mental well-being.

#### Acute psychedelic effects :

One day after the psychedelic session, measures of acute psychedelic effects were assessed. These included: (1) the Ego-Dissolution Inventory (EDI),^43^ a measure reflecting the loss of a subjective experience of the self, which is typically induced by psychedelics, that has participants rate 10 items on a 0–100 scale; (2) the Mystical Experience Questionnaire (MEQ),^44^ a 30-item 6-point Likert scale measuring facets of mystical-type and peak experiences; (3) the Challenging Experience Questionnaire (CEQ),^45^ a 26-item 6-point Likert scale assessing difficult responses to the drug, such as fear, paranoia, and physiological alterations; (4) the Emotional Breakthrough Inventory (EBI),^18^ a six-item scale assessing emotional release and resolution of past trauma; and (5) the Communitas Scale (COMS) a 8-item questionnaire assessing acute relational experiences of togetherness and collective joy during psychedelic group sessions.^34^

#### Post-acute mediators:

One day post-retreat (on the day after leaving the ceremony location), participants completed: (1) the Psychological Insight Scale (PIS), assessing the degree to which the psychedelic experience was perceived as psychologically insightful via six 0–100 visual analogue scale items, and (2) a version of the communitas scale (COMS_PR_), modified to assess relational experiences during the overall retreat, as opposed to the substance session only.

### Statistical Analyses

Three mixed linear effects models, each including a random intercept, were used to assess changes in WEMWBS scores from baseline to two weeks, four weeks, and six months after the psychedelic session: a first model in OA, including only time point as a fixed effect, a second model in OA to which baseline demographic characteristics and their interaction with time points were added, and a third model comparing longitudinal changes in WEMWBS across OA and matched YA by including the interaction of time points and age group in the fixed portion of the model. Two-tailed paired *t*-tests were used to further assess significant WEMWEBS changes from baseline to each of the later time points in the whole OA sample and in a subsample of OA with a self-reported history of psychiatric diagnosis.

Next, we used MANOVA and ANOVA models to compare the intensity of subjective acute psychedelic effects across the OA and YA groups. To explore potential pairwise dependency effects introduced by 1:1 matching, results of both paired and unpaired t-tests are reported in Supplementary Table S2. Additionally, a fourth mixed linear model with OA and matched YA was used to assess a three-way interaction between subjective acute psychedelic effects, age group, and changes in WEMWBS, aiming to expose age-related differences in salutogenic mechanisms. Pseudo-standardized regression coefficients *β* were calculated for this model to facilitate interpretability of the findings with *β* coefficients >0.1, 0.3, and 0.5, respectively indicating weak, medium, or strong associations. Additional pairwise Pearson’s correlations between WEMWBS change scores from baseline to the two- and four-week endpoint and subjective acute and post-acute psychedelic effects scores were calculated separately for OA and YA, with the purpose to illustrate three-way interaction results in a simplified manner. A significance level of p <0.05 was applied to all statistical tests. Where applicable in case of multiple comparisons, both uncorrected and Bonferroni-corrected levels of significance are reported.

## RESULTS

### Demographic Information

A total of 882 participants signed up for the study, out of whom 819 provided baseline information. Among 106 participants that reported ≥60 years of age, 62 had completed the baseline as well as the 24 h post-session survey, yielding the final OA sample analyzed in our study. Among these 62 OA, 53 completed the post-retreat questionnaire, evaluating experiences across the entire retreat period; 44 completed the two-week, 61 completed the four-week, and 23 completed the six-months endpoints. From a total of 430 adults with age <60 years who completed at least the baseline and the 24 h post-session survey, a set of 62 matched YA were selected using nearest k-neighbor matching (Table 1). For an overview of demographic information in the full YA sample, see Supplementary Table S1.

The mean age in the identified 62 OA was 65.1 years (SD = 4.02; range = 60–75) and exactly half (31/62, 50.0%) were male. A majority (43/62, 68.5%) of OA had a master’s degree or higher, no history of diagnosed mental illness (46/62, 74.2%), and no prior experiences with psychedelics (35/62, 56.5%). Among OA who indicated psychiatric diagnoses, the most common were major depressive disorder (10/16) and anxiety disorder (9/16); alcohol dependence and ADHD were indicated by two individuals, respectively; personality, bipolar, and eating disorders by one person each. 59 OA attended psilocybin mushroom or truffle sessions (57 of which took place at retreat centers in the Netherlands or Jamaica), while three individuals indicated ayahuasca as the used psychedelic. A sample of 62 YA (mean age in years [SD] = 46.5 (10); range = 24–59) was selected via nearest k-neighbor matching for comparison purposes.

### Post-Psychedelic Mental Health Improvements

**FIGURE 1.**
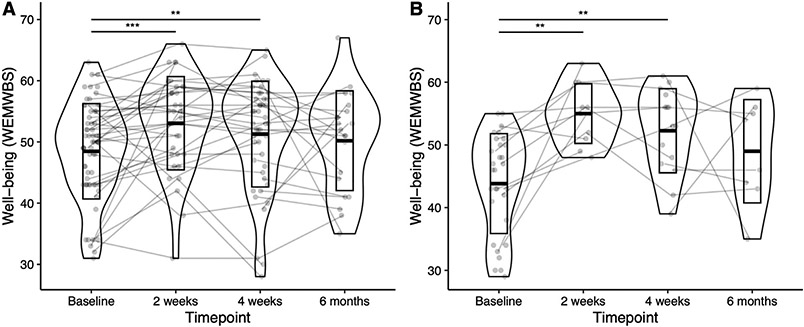
Mental well-being increases in OA following a psychedelic group session. Violinplots showing the distribution of WEMWBS scores in all OA (A) and OA with a lifetime psychiatric diagnosis only (B) at each time point, with lines reflecting individual trajectories. Significance values derived from mixed linear regression models (N_A_ = 62, N_B_ = 16). OA: older adults; WEMWBS: Warwick-Edinburgh Mental Wellbeing Scale; **p <0.01, ***p <0.001.

A mixed effects linear regression model revealed WEMWBS increases in OA following the psychedelic session (Fig. 1A). An average increase of four points on the WEMWBS was found at the two-week endpoint (*B* = 4.09, 95% CI [1.87, 6.31], p < 0.001); this remained a three-point increase at the four-week endpoint (*B* = 3.05, 95% CI [1.04, 5.06], p = 0.004), indicating meaningful improvements in well-being. At six months post dosing, well-being scores were still nominally elevated by 1.7 units, which did not, however, reach significance (*B* = 1.72, 95% CI [−1.00, 4.44], p = 0.22). Paired *t*-tests comparing endpoint to baseline scores confirmed this pattern, with significant well-being increases at two weeks (p = 0.006, Cohen’s d=0.48, *t* (34)=−2.87) and four weeks (p = 0.004, Cohen’s d = 0.44 *t* (45) = −3.01) but not at six months (p = 0.63, Cohen’s d = 0.10, *t* (20) = −0.48).

Having established that well-being improves in OA following psychedelic group sessions, we next investigated whether any individual demographic characteristics predicted post-psychedelic changes in mental well-being. We conducted a mixed effects linear regression model including age, gender, education, extent of prior experience with psychedelic substances, expectations on the beneficial effects of psychedelics, and history of mental illness as predictors. This model revealed only an interaction of history of mental illness with time (*B* = 6.62, 95% CI [1.53, 11.71], p = 0.019 at two weeks; *B* = 5.69, 95% CI [1.14, 9.94], p = 0.016 at four weeks), indicating that well-being increased more drastically in OA reporting a lifetime psychiatric diagnosis (Table 2). Paired *t*-tests within the subsample of OA with a psychiatric diagnosis (Fig. 1B) revealed significantly increased WEMWBS scores at two weeks (p = 0.007, Cohen’s d = 1.02, *t* (10) = −3.42), with an average increase of 9.4 points at four weeks (p = 0.004, Cohen’s d = 0.89 *t* (14) = −3.44) but not at six months (p = 0.48, Cohen’s d = 0.10, *t* (7) = −0.74).

We last aimed to assess whether changes in OA were comparable to those observed in YA. We conducted a mixed effects linear regression model with the OA and matched YA samples, which revealed no significant interactions between age group and time, indicating that well-being improvements in OA and YA were statistically indistinguishable in this sample (*B* = −2.36, 95% CI [−5.48, 0.76], p = 0.14 and *B* = −2.55, 95% CI [−5.53, 0.42], p = 0.097, for two weeks and four weeks post-dosing, respectively; Supplementary Fig. S1).

### Comparing Subjective Psychedelic Effects in OA and YA

**FIGURE 2.**
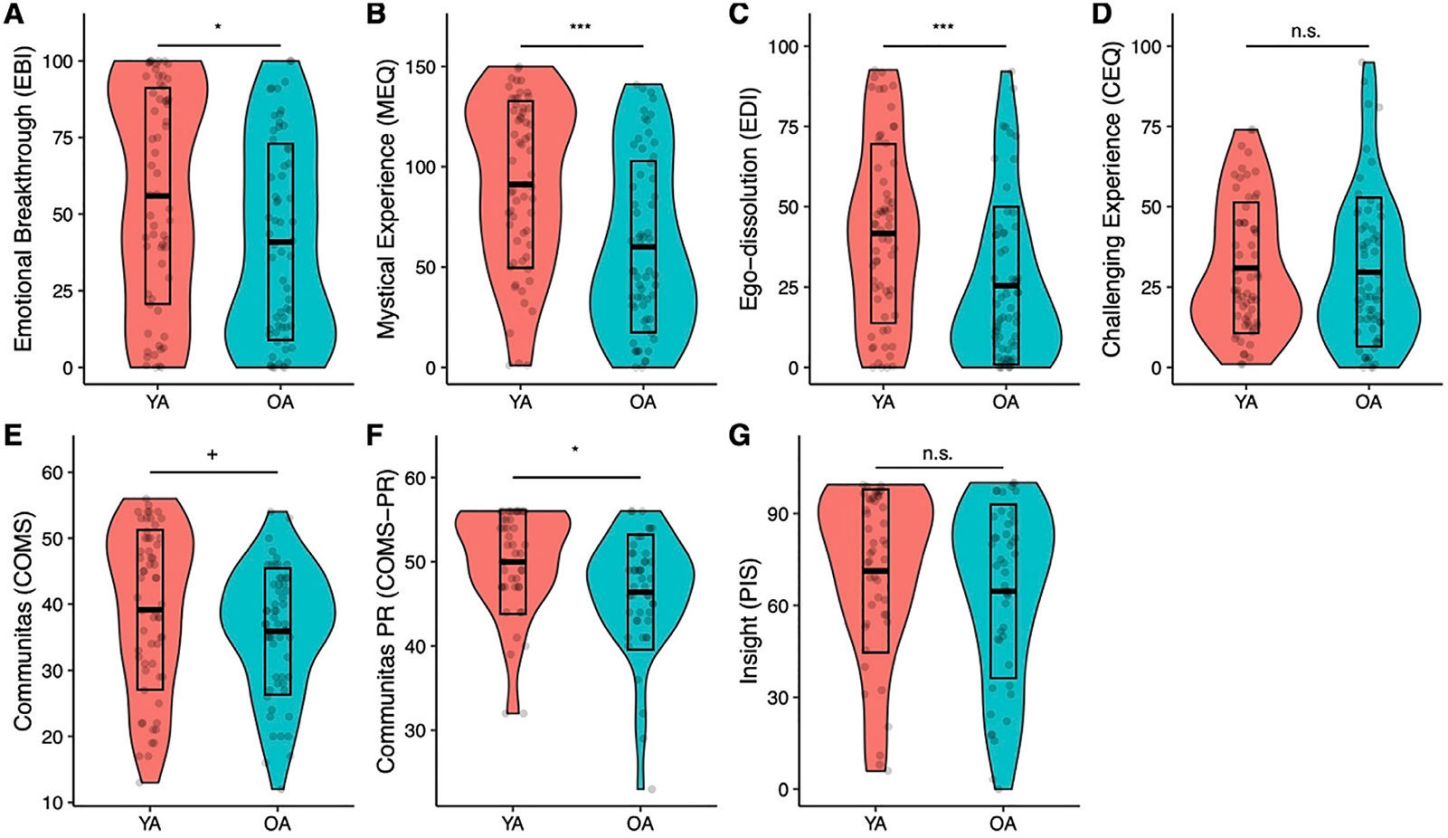
Violin plots showing intensity ratings of subjective psychedelic effects. MANOVAs revealed attenuated acute (A–E, df = 1,111) and post-acute (F–G, df = 1,86) subjective effects in OA (≥60 years) compared to YA. OA: Older adults; PR: post-retreat; YA: Younger adults. ^+^p <0.01, *p <0.05; **p <0.01; ***p <0.001.

A MANOVA comparing ratings of acute subjective effects (EBI, MEQ, EDI, CEQ, COMS) between OA and YA revealed significant differences between the groups (Pillais’ Trace = .14, *F*(5,110) = 3.72, p = 0.004). Follow-up ANOVAs were then conducted (Fig. 2), revealing significantly lower intensity scores for OA on all tests included subjective effects measures, except for the CEQ (*F* [1,114] = 0.61, *d* = 0.06, p = 0.43) and only at trend level for the COMS (*F* [*1,114*] = 2.91, *d* = 0.30, p = 0.09), suggesting that OA experienced overall less intense subjective psychedelic effects compared to YA. The differences in mean scores between OA and YA were greatest for the EDI (48.6%, M = 25.4 vs 41.7, *F* [*1,114*] = 13.75, d = 0.62, p < 0.001) and the MEQ (39.1%, M = 60.1 vs M = 89.3, *F* [*1,114*] = 17.65, *d* = 0.74, p < 0.001), followed by the EBI (31.0%, M = 40.9 vs M = 55.9, *F* [*1,114*] = 5.44, *d* = 0.45, p = 0.02). Only differences on the MEQ and EDI were significant also after five-fold Bonferroni-correction (p < 0.001 and p = 0.002, respectively)

In a second MANOVA, post-acute effects, including insights (PIS) and Communitas experienced across the retreat as a whole (COMS-PR) were also found to be different by age group (Pillais’ Trace = .07, *F* (2,86) = 3.11, p = 0.05). Follow-up ANOVAs revealed this difference to be based on COMS-PR scores, which were significantly lower in OA (7.5%, M = 46.39 vs 49.98, *F* (1,87) = 6.30, *d* = 0.55, p = 0.01), while PIS scores were not significantly different (M = 46.39 vs 43.98, *F* (1,87) = 0.27, *d* = 0.24, p = 0.60).

### Differential Mechanisms Predicting Well-Being Changes in OA and YA

We subsequently explored whether age group-dependent acute and post-acute subjective psychedelic effects predicted long-term well-being outcomes via a mixed effects linear regression model including three-way interaction terms between age group, time, and each acute predictor variable. To prevent multicollinearity issues, variance inflation factors (VIF) for each included predictor variable were calculated, resulting in the removal of the MEQ from the model (VIF = 3.7) based on the generally accepted VIF cut-off of 2.5.^46^ After excluding MEQ, the highest VIF was found for EBI but beneath the established cut-off (VIF = 2.1). The resulting model revealed a significant and large negative three-way interaction between EBI, older age group, and post-psychedelic endpoints at two-(*β* = −0.78, 95% CI [−1.43, −0.13], p = 0.02) and four-weeks (*β* = −0.80, 95% CI [−1.46, −0.15], p = 0.02), indicating that emotional breakthrough experiences contributed less to improved well-being in OA compared to YA (Table 3). Furthermore, a very large, albeit only marginally significant positive three-way interaction was detected for post-retreat COMS scores, OA, and the four-week endpoint, suggesting that relational experiences of sharing and togetherness across the retreat may have played a larger role for predicting improved well-being in OA when compared to YA (*β* = 2.04, 95% CI [−0.21, 4.28], p = 0.08).

**FIGURE 3.**
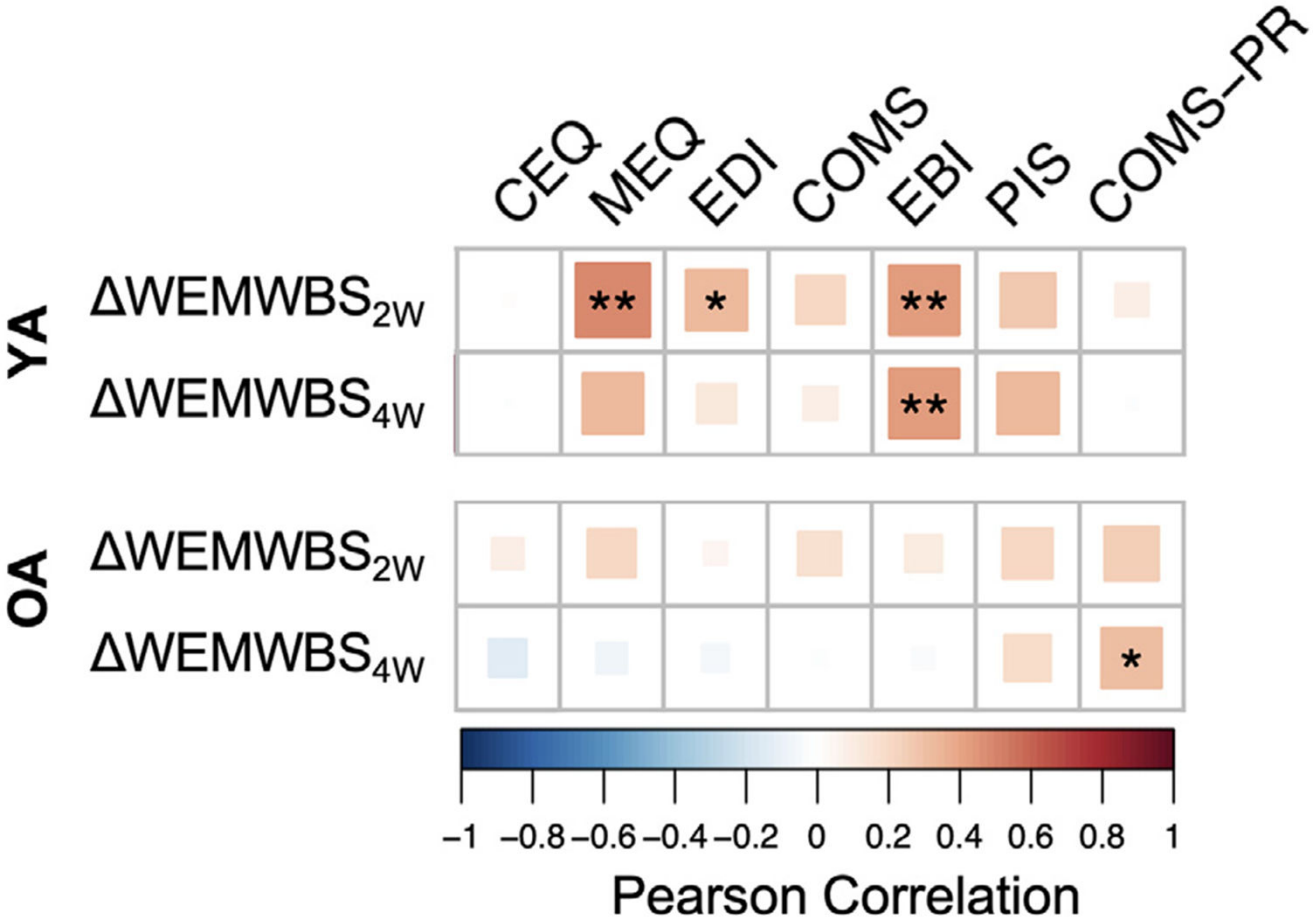
Pearson correlation heatmap showing associations between subjective psychedelic effects and changes in well-being from baseline to two weeks (2W) and four weeks (4W) after a psychedelic group session. CEQ: Challenging Experience Questionnaire; COMS: Communitas Scale; EBI: Emotional Breakthrough Inventory; EDI: Ego-Dissolution Inventory; MEQ: Mystical Experience Questionnaire; OA: Older adults; PR: Post-retreat; YA: Younger adults. *p <0.05; **p <0.01.

Correlation analyses between predictors and well-being change scores (Fig. 3) further illustrate these relationships: EBI was moderately associated with well-being change scores at four-weeks in YA (r [35] = .43, p = 0.008), but only negligibly in OA (r [46] = −.04, p = 0.79), while post-retreat COMS-PR was positively associated with well-being change scores at four-weeks in OA (r [27] = .37, p = 0.03) but not in YA (r [34] = .01, p = 0.94). Interestingly, none of the other acute and post-acute variables that significantly correlated with well-being changes in YA were shown to significantly correlate with well-being changes in OA. Comprehensively, these findings suggest that the psychedelic experience fundamentally differs between OA and YA indicating a unique role for psychosocial experiences in the older group.

## DISCUSSION

In this prospective study, we investigated the effects of naturalistic guided psychedelic group sessions on OA’s well-being by leveraging an opportunity sample of 62 participants aged 60 years or older attending self-initiated psychedelic ceremonies or retreats. Analyses revealed clinically meaningful improvements in well-being in OA at two and four weeks following a psychedelic group session, in line with prior naturalistic studies in YA.^34,47-49^ Interestingly, this was the case despite lower acute subjective effects scores in the OA sample, indicating that differential salutogenic mechanisms may be at play in this age group. This exploratory hypothesis was partially confirmed through regression and correlational analyses suggesting a primacy of relational mechanisms, as opposed to classic intrasubjective psychedelic effects in OA attending psychedelic group sessions.

Among baseline and demographic variables predicting well-being increases in OA, only the presence of a psychiatric diagnosis showed significant effects. This finding was stable also when controlling for expectation effects, a hypothesized confounder in psychedelic trials^50^ and is in line with the transdiagnostic utility of psychedelic treatments for a number of mental health disorders,^51-53^ including major depression, alcohol-use disorder, and anorexia nervosa. Indeed, resilience to expectancy is consistent with recent research that failed to support its influence in driving therapeutic response to psilocybin therapy for depression,^54^ implying a substantive direct therapeutic action. Outside regulated clinical trial settings, the structured, user-reviewed services offered by retreat centers might have particular appeal to OA when compared to individual use (e.g., at-home). OA may have less access to or tend to avoid the acquisition of scheduled substances over the black market, may have greater psychological needs for safety, structure and social contact,^55^ and the economical means to afford the often high financial cost of psychedelic retreats or ceremonies.

Crucially, at least based on the limited present sample, clinically relevant improvements in mental health in OA were not significantly different from those found in YA, matched to account for several demographic factors including higher OA well-being at baseline, a common finding in the literature.^2^ For example, elevated baseline well-being levels in OA are in accordance with representative population level studies showing that in wealthy English-speaking countries, happiness and hedonic experiences are lowest around ages 45–54 and tend to increase with age, following an inverted U-shape.^56,57^

The observed return of well-being levels to baseline at the six-months follow-up time point in OA is in contrast with prior studies showing long-term mental health improvements following psychedelic-assisted psychotherapy reviewed in.^58^ Two prior naturalistic studies in YA have also found sustained two-year increases in protective psychological traits such as resilience and mindfulness,^59^ or nature relatedness,^60^ although, similar to the present study, affective measures of well-being have thus far been shown to remain increased only at nominal, non-significant levels.^59^ The conditions under which psychedelic-induced salutogenesis remains stable therefore remains a critical unanswered question, considering that in clinical studies, improvements appear to remain significant for months to years following treatment.^61-64^

Our study revealed attenuated acute psychedelic effects and different salutogenic mechanisms in OA when compared to YA. This is of clinical importance, since current models of psychedelic function propose that the acute psychedelic effects are key mediators of mental health improvements.^16,65-68^ In contrast to prior controlled research reporting challenging experiences to be negatively correlated with age,^69-71^ the OA group in the present sample showed lower acute effects scores on all metrics *except* for challenging experiences. One potential reason for this apparent discrepancy may be the overall younger age (means ranging from 27–36) and lack of participants aged 60 or above in the abovementioned controlled studies. It is thus possible that the intensity of challenging experiences under psychedelics peaks among the younger distribution of YA and remains stable after a certain age, pointing to sample diversity as a key strength of naturalistic studies such as this one. Overall, the finding of attenuated psychedelic intensity scores in OA can be seen as a positive signal towards the psychological safety profile of psychedelic interventions in this age group, and it is reassuring that well-being improvements were detected in spite of the often discussed therapeutic function of ego disturbances in psychedelic drug action.^72^

Nonetheless, the absence of any significant correlations between acute psychedelic effects and long-term changes in OA represents an interesting contradiction to previous work showing that the quality of the acute experience constitutes a key predictor of psychedelic-induced changes in well-being.^16-19,34,73,74^ In contrast, only the experience of Communitas rated in reference to the entire retreat—not just the psychedelic session was associated with well-being changes in OA. The strong, albeit only marginally significant 3-way interaction including age group and retreat-Communitas suggests that OA might benefit from psychedelics for different reasons than YA, greater relevance being given to the experience of togetherness and social bonds that can occur in group settings than to the individual, intrapersonal experience. The witnessing of other participants’ vulnerability and the resulting emotional intimacy generated through sharing rounds before and after dosing sessions might be particularly impactful to OA, for whom social contact, especially with nonfamily members, is known to decrease.^75^ Indeed, from the present data it is unclear to what extent the consumption of the psychedelic substance itself would have even been necessary for OA to experience the detected psychological benefits. Future research should thus further explore the details of psychotherapeutic and group activities taking place at psychedelic retreats, and their psychological benefits for participants, as well as the validity of instruments assessing the overall experience in OA. Conceivably, the psychedelic session itself could be seen as a non-essential part, primarily providing the context for an intimate and intergenerational group-based intervention with the potential to tackle the negative emotional and cognitive health consequences of social isolation in the elderly.^76,77^

The present findings of reduced acute psychedelic effects and increased importance of social connections may relate to the consolidation of “emotional landscapes” in OA.^2^ Our findings are in line with Carstensen’s^1^ Socioemotional Selectivity Theory posing that OA optimize emotional experiences to prioritize meaningful social connections and foster positive experiences and emotional satisfaction. Intriguingly, reduced acute psychedelic effects in OA may mechanistically also relate to age-dependent reductions in cortical serotonin receptor density, which is most pronounced for the 2A receptor,^78^ the primary target of psychedelics.^14,79^

Several limitations need to be considered when interpreting our findings. Most importantly, the context of psychedelic use in the present sample was limited to ceremony and retreat settings, raising the question whether well-being improvements, attenuated acute psychedelic effects, and greater importance of psychosocial mechanisms detected in the current sample would also occur in other naturalistic or controlled psychotherapeutic settings. Replications in larger and more representative samples will therefore be crucial to further explore the effects of psychedelic on the elderly outside psychedelic ceremony and retreat settings, and in samples less biased towards white, highly educated participants. Controlled laboratory studies administering psychedelics to OA will potentially be able to clarify the role of acute psychedelic effects in environments that do not provide the psychosocial group benefits present at psychedelic retreats. Additional limitations include the inaccurate qualitative assessment of psychedelic dose, as well as possible co-use of other substances common in naturalistic samples^80,81^ which was not controlled for in the present study. Furthermore, the potential of systematic biases through attrition effects constitutes another limitation related to the remote nature of this survey study, although prior research has shown that attrition in prospective psychedelic surveys follows similar patterns as in other fields.^82^

## CONCLUSIONS AND FUTURE DIRECTIONS

Echoing previous observational studies in YA and clinical trials, our findings show that psychedelic group sessions can induce rapid and sustained benefits on OA’s well-being, including in those with a history of a psychiatric illness. However, compared to their younger counterparts, OA reported an overall attenuated intensity of acute psychedelic effects, including ego dissolution, mystical, and emotional breakthrough experiences. Although additional replication studies will be necessary, our findings instead indicate that psychosocial experiences, as encountered in group and ceremonial settings, may be particularly valuable for OA. Considering the vast underrepresentation of OA in contemporary psychedelic trials, we hope that this first inquiry into the effects of psychedelics in OA will spur further investigation into the clinical and long-term utility of psychedelics for issues prevalent in the older population.

## Supplementary Material

supplement

## Figures and Tables

**TABLE 1.** Demographic Characteristics of the Study Samples

	OA (N = 62)	YA Matched (N = 62)
Age in years		
Mean (SD)	65.1 (4.02)	46.5 (10.0)
Median (min, max)	64.0 (60.0, 75.0)	49.0 (24.0, 59.0)
Gender		
Male	31 (50.0%)	31 (50.0%)
Female	31 (50.0%)	31 (50.0%)
Other	-	-
Education/degrees		
None	1 (1.6%)	0 (0%)
High school	3 (4.8%)	2 (3.2%)
Technical degree	4 (6.5%)	5 (8.1%)
College diploma	11 (17.7%)	18 (29.0%)
Master	19 (30.6%)	30 (48.4%)
PhD/MD/Law degree	24 (38.7%)	7 (11.3%)
Psychiatric diagnoses		
Yes	16 (25.8%)	15 (24.2%)
No	46 (74.2%)	47 (75.8%)
Psychedelic use #		
Never	35 (56.5%)	28 (45.2%)
1–5 times	15 (24.2%)	10 (16.1%)
>5 times	12 (29.3%)	24 (38.7%)
WEMWBS baseline		
Mean (SD)	48.5 (7.79)	48.8 (9.79)
Median (min, max)	50.0 (31.0, 63.0)	50.5 (20.0, 70.0)
Psychedelic dose		
Mean (SD)	2.10 (0.646)	2.13 (0.586)
Median (min, max)	2.00 (1.00, 3.00)	2.00 (1.00, 3.00)

OA: Older Adults; SD: Standard Deviation; WEMWBS: Warwick-Edinburgh Mental Wellbeing Scale; YA: Younger Adults.

**TABLE 2.** Main Effects and Two-Way Interactions From a Mixed Linear Regression Model (N = 62) of Baseline and Demographic Variables on Wellbeing Across Time in OA

Fixed Effects				
Term	*B*	SE	t-Value	p
Intercept	19.28	17.99	1.071	0.28
2-week endpoint	9.77	18.42	0.530	0.60
4-week endpoint	15.74	16.39	0.960	0.34
Age	0.39	0.26	1.502	0.14
Gender (f)	−1.44	2.12	−0.681	0.50
Psychedelic use	0.61	0.67	0.914	0.36
Psychiatric history	−3.00	2.45	1.207	0.23
Expectations	0.01	0.06	0.258	0.80
Age: 2-week endpoint	0.00	0.27	−0.016	0.99
Age: 4-week endpoint	−0.17	0.23	−0.734	0.47
Gender (f): 2-week endpoint	−0.82	2.21	−0.373	0.71
Gender (f): 4-week endpoint	2.46	1.96	1.259	0.21
Psychedelic use: 2-week endpoint	−0.70	0.65	−1.083	0.28
Psychedelic use: 4-week endpoint	0.06	0.57	0.097	0.92
Psychiatric history: 2-week endpoint	−6.62	2.77	−2.388	**0.02**
Psychiatric history: 4-week endpoint	−5.69	2.32	−2.457	**0.02**
Expectations: 2-week endpoint	0.02	0.06	0.403	0.69
Expectations: 4-week endpoint	0.02	0.05	0.4000	0.69
	Random effects V		SD	
Participant (intercept)	43.54		6.599	
	Model fit Marginal		Conditional	
R^2^	11.7%		70.4%	

f: Female; SD: Standard Deviation; SE: Standard Error; V: Variance. Values in bold are significant at p<.05.

**TABLE 3.** Three-Way Interactions From a Mixed Linear Regression Model (N = 82) Showing Differential Salutogenic Mechanisms for OA and YA, Including Pseudo-Standardized Regression Coefficients (β)

Fixed Effects				
Term	β	SE	t-Value	p
EDI: Age 60+: 2-week endpoint	0.05	0.21	0.25	0.80
EDI: Age 60+: 4-week endpoint	0.05	0.24	0.217	0.83
EBI: Age 60+: 2-week endpoint	−0.78	0.33	−2.354	**0.02**
EBI: Age 60+: 4-week endpoint	−0.8	0.33	−2.416	**0.02**
CEQ: Age 60+: 2-week endpoint	0.18	0.23	0.788	0.43
CEQ: Age 60+: 4-week endpoint	0.2	0.23	0.855	0.39
COMS: Age 60+: 2-week endpoint	0.38	0.53	0.727	0.47
COMS: Age 60+: 4-week endpoint	−0.14	0.58	−0.24	0.81
COMS_PR_: Age 60+: 2-week endpoint	−0.04	1.05	−0.04	0.97
COMS_PR_: Age 60+: 4-week endpoint	2.04	1.15	1.793	0.08
PIS: Age 60+: 2-week endpoint	−0.06	0.39	−0.16	0.87
PIS: Age 60+: 4-week endpoint	−0.09	0.38	−0.234	0.82
	Random effects V		SD	
Participant (intercept)	36.74		6.062	
	Model fit Marginal		Conditional	
R^2^	26.9%		70.0%	

For a full tabulation of unstandardized effects, including main effects and lower-order interactions, see Supplementary Table S3. CEQ: Challenging Experience Questionnaire; COMS: Communitas Scale; EBI: Emotional Breakthrough Inventory; EDI: Ego-Dissolution Inventory; PR: Post-Retreat; PIS: Psychological Insight Scale; SD: Standard Deviation; SE: Standard Error; V: Variance. Values in bold are significant at p<.05.

## Data Availability

Raw data can be shared from the corresponding author following reasonable request.

## Highlights

- What is the primary question addressed by this study?—The question addressed by the study must limited to only one sentence.
  Psychedelic substances have gained increased interest as experimental therapeutics for a variety of neuropsychiatric disorders, yet evidence on the effects of psychedelics in older adults remains scarce.
- What is the main finding of this study?—The finding must be limited to two sentences.
  Older adults reported well-being improvements similar to a matched sample of younger adults following participation in a psychedelic retreat, despite significantly attenuated acute subjective intensity scores.
  In contrast to the existing literature based largely on younger adults, well-being changes in older adults were not predicted by acute psychedelic effects, but rather by interpersonal experiences of togetherness ("communitas") during group activities across the entire retreat.
- What is the meaning of the finding?—The meaning of the finding must be limited to one sentence.
  These findings bear significance for benefit-maximization and harm reduction practices for naturalistic psychedelic use in older adults, as well as clinical trial design, providing some initial evidence to address the critical knowledge gap regarding the effects of psychedelic substances in older adults.

## DATA STATEMENT

The data has not been previously presented orally or by poster at a scientific meeting.
