# Supplementary material for: Effects of Psychedelics in Older Adults: A Prospective Cohort Study
Source: Am J Geriatr Psychiatry. Author manuscript; Available in PMC 2024 Sep 1. (PMC11316630; doi:10.1016/j.jagp.2024.05.007)
Supplement: supplement [file NIHMS2002949-supplement-supplement.docx]

**Supplementary Material:**

**Effects of psychedelics in older adults: A prospective cohort study**

|  | YA unmatched (N=430) | YA matched (N=62) | OA (N=62) |
| --- | --- | --- | --- |
| **Age in years** |  |  |  |
| Mean (SD) | 41.4 (9.8) | 46.5 (10.0) | 65.1 (4.02) |
| Median [Min, Max] | 41.0 [21.0, 59.0] | 49.0 [24.0, 59.0] | 64.0 [60.0, 75.0] |
| **Gender** |  |  |  |
| Male | 239 (55.6%) | 31 (50.0%) | 31 (50.0%) |
| Female | 188 (43.7%) | 31 (50.0%) | 31 (50.0%) |
| Other | 3 (07%) | - | - |
| **Education/degrees** |  |  |  |
| None | 3 (0.7%) | 0 (0%) | 1 (1.6%) |
| High school | 20 (4.7%) | 2 (3.2%) | 3 (4.8%) |
| Technical degree | 32 (7.4%) | 5 (8.1%) | 4 (6.5%) |
| College diploma | 141 (32.8%) | 18 (29.0%) | 11 (17.7%) |
| Master | 152 (35.3%) | 30 (48.4%) | 19 (30.6%) |
| PhD/MD/Law degree | 82 (19.1%) | 7 (11.3%) | 24 (38.7%) |
| **Psychiatric diagnoses** |  |  |  |
| Yes | 146 (34.0%) | 15 (24.2%) | 16 (25.8%) |
| No | 284 (66.0%) | 47 (75.8%) | 46 (74.2%) |
| **Psychedelic use #** |  |  |  |
| Never | 169 (39.3%) | 28 (45.2%) | 35 (56.5%) |
| 1-5 times | 143 (33.3%) | 10 (16.1%) | 15 (24.2%) |
| > 5 times | 118 (27.4%) | 24 (38.7%) | 12 (29.3%) |
| **WEMWBS baseline** |  |  |  |
| Mean (SD) | 45.1 (9.03) | 48.8 (9.79) | 48.5 (7.79) |
| Median [Min, Max] | 46.0 [20.0, 70.0] | 50.5 [20.0, 70.0] | 50.0 [31.0, 63.0] |
| **Psychedelic dose** |  |  |  |
| Mean (SD) | 2.33 (0.632) | 2.13 (0.586) | 2.10 (0.646) |
| Median [Min, Max] | 2.00 [1.00, 3.00] | 2.00 [1.00, 3.00] | 2.00 [1.00, 3.00] |
| **Supplementary Table S1. Demographic characteristics of the excluded YA sample.** OA=older adults; SD: Standard Deviation; WEMWBS: Warwick-Edinburgh Mental Wellbeing Scale; YA: younger adults | | | |

| Outcome | YA | OA | independent | | paired | |
| --- | --- | --- | --- | --- | --- | --- |
|  | M (SD) | M (SD) | t | p | t | p |
| MEQ | 89.3 (41.6) | 60.1 (42.6) | -4.09 | **<.001** | -4.39 | **<.001** |
| EDI | 41.7 (27.9) | 25.4 (24.5) | -3.43 | **<.001** | -3.57 | **<.001** |
| EBI | 55.9 (35.3) | 40.9 (32.0) | -2.47 | **.015** | -2.61 | **.011** |
| CEQ | 30.98 (20.3) | 29.7 (23.2) | -0.34 | .74 | -0.44 | .66 |
| COMS | 39.2 (12.1) | 35.9 (9.6) | -1.64 | .10 | -1.09 | .27 |
| COMS_PR_ | 50.0 (6.2) | 46.4 (6.8) | -2.62 | **.01** | -3.68 | **<.001** |
| PIS | 77.4 (26.6) | 64.6 (28.3) | -1.16 | .24 | -0.65 | .52 |
| **Supplementary Table S2. Age-related differences in acute experience intensity ratings.** Mean (M), standard deviation (SD) and results of paired and independent samples t-tests are shown for N=62 adults ≥60 years (OA) and N=62 baseline-matched adults <60 years (YA). | | | | | | |

| Term | B | SE | t-value | p |
| --- | --- | --- | --- | --- |
| Intercept | 36.7 | 1.47 | 2.50 | **.01** |
| Age 60+ | 1.34 | 17.49 | 0.08 | .94 |
| 2-week endpoint | -3.11 | 14.30 | -0.22 | .83 |
| 4-week endpoint | 16.27 | 14.43 | 1.13 | .26 |
| EDI | 0.00 | 0.01 | -0.58 | .56 |
| EBI | -0.02 | 0.01 | -1.43 | .16 |
| CEQ | -0.06 | 0.07 | -0.86 | .39 |
| COMS | 0.14 | 0.15 | 0.93 | .35 |
| COMS_PR_ | 0.16 | 0.31 | 0.53 | .60 |
| PIS | 0.02 | 0.01 | 1.95 | .05 |
| Age 60+:2-week endpoint | 2.46 | 19.15 | 0.13 | .90 |
| Age 60+:4-week endpoint | -22.89 | 17.66 | -1.30 | .20 |
| EDI:Age 60+ | 0.01 | 0.01 | 0.90 | .37 |
| EDI:2-week endpoint | 0.00 | 0.01 | -0.22 | .82 |
| EDI:4-week endpoint | 0.00 | 0.01 | 0.01 | .99 |
| EBI:Age 60+ | 0.02 | 0.01 | 1.66 | .10 |
| EBI:2-week endpoint | 0.03 | 0.01 | 2.63 | **.01** |
| EBI:4-week endpoint | 0.02 | 0.01 | 2.13 | **.04** |
| CEQ:Age 60+ | 0.02 | 0.10 | 0.25 | .81 |
| CEQ:2-week endpoint | -0.12 | 0.07 | -1.57 | .12 |
| CEQ:4-week endpoint | -0.05 | 0.08 | -0.62 | .54 |
| COMS:Age 60+ | -0.11 | 0.22 | -0.49 | .62 |
| COMS:2-week endpoint | -0.36 | 0.16 | -2.28 | **.02** |
| COMS:4-week endpoint | -0.18 | 0.16 | -1.12 | .26 |
| COMS_PR_:Age 60+ | 0.05 | 0.37 | 0.13 | .90 |
| COMS_PR_:2-week endpoint | 0.27 | 0.30 | 0.89 | .38 |
| COMS_PR_:4-week endpoint | -0.32 | 0.31 | -1.06 | .29 |
| PIS:Age 60+ | -0.03 | 0.02 | -2.01 | **.05** |
| PIS:2-week endpoint | 0.01 | 0.01 | 0.72 | .48 |
| PIS:4-week endpoint | 0.01 | 0.01 | 1.30 | .20 |
| EDI:Age 60+:2-week endpoint | 0.03 | 0.12 | 0.25 | .80 |
| EDI:Age 60+:4-week endpoint | 0.02 | 0.11 | 0.217 | .83 |
| EBI:Age 60+:2-week endpoint | -0.22 | 0.10 | -2.354 | **.02** |
| EBI:Age 60+:4-week endpoint | -0.21 | 0.09 | -2.416 | **.02** |
| CEQ:Age 60+:2-week endpoint | 0.08 | 0.11 | 0.788 | .43 |
| CEQ:Age 60+:4-week endpoint | 0.09 | 0.10 | 0.855 | .39 |
| COMS:Age 60+:2-week endpoint | 0.18 | 0.25 | 0.727 | .47 |
| COMS:Age 60+:4-week endpoint | -0.06 | 0.24 | -0.24 | .81 |
| COMS_PR_:Age 60+:2-week endpoint | -0.02 | 0.38 | -0.04 | .97 |
| COMS_PR_:Age 60+:4-week endpoint | 0.68 | 0.38 | 1.793 | **.08** |
| PIS:Age 60+:2-week endpoint | 0.00 | 0.02 | -0.16 | .87 |
| PIS:Age 60+:4-week endpoint | 0.00 | 0.02 | -0.234 | .82 |
| **Supplementary Table S3.** Main effects, two-way and three-way interactions showing differential salutogenic mechanisms for older and younger adults*.* CEQ: Challenging Experience Questionnaire; COMS: Communitas Scale; EBI: Emotional Breakthrough Inventory; EDI: Ego-Dissolution Inventory; PR: Post-retreat; PIS: Psychological Insight Scale; SE: Standard Error. | | | | |

| 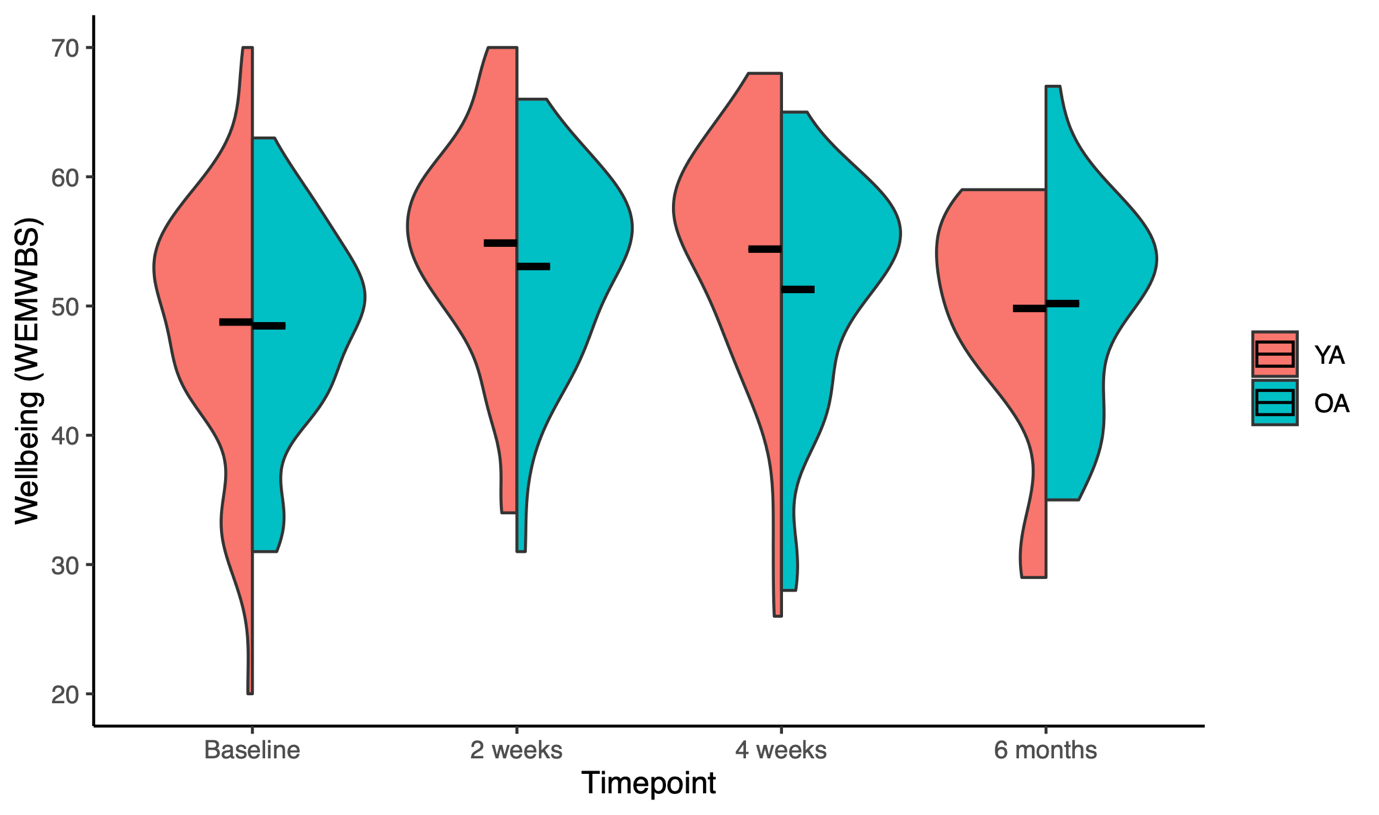 |
| --- |
| **Supplementary Figure S1. Mental well-being increases in OA and YA following a psychedelic group session.** Violinplots showing the distribution of WEMWBS scores in OA and baseline-matched YA at each time point. Bars represent mean scores. OA: older adults; WEMWBS: Warwick-Edinburgh Mental Wellbeing Scale; YA: younger adults. |
